# Supplementary material for: Integrating genomic structural equation modeling and experimental validation to unravel the genetic basis of male genital lichen sclerosus
Source: Front Immunol. 2026 Jul 16;17:1857945. doi: 10.3389/fimmu.2026.1857945 (PMC13435250; doi:10.3389/fimmu.2026.1857945)
Supplement: Supplementary file 6 [file SupplementaryFile5.zip › result/fuma_PSYCH_factor_sCCA3_focus.chr2.chr2.215573795.217715661.pop1.pdf]

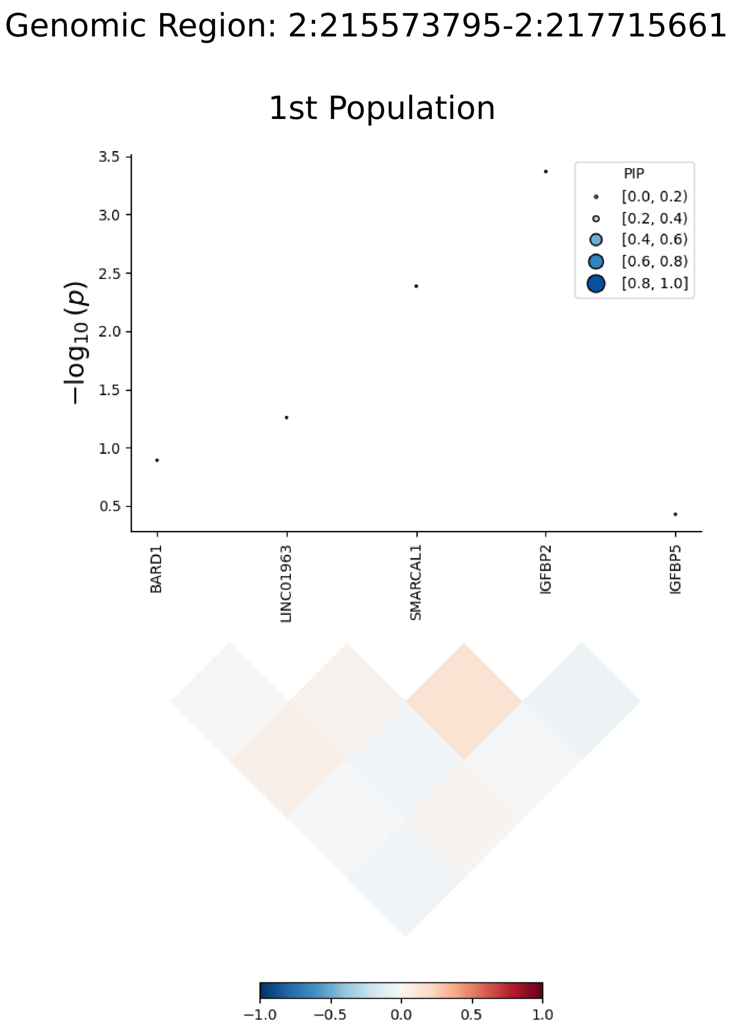
Genomic Region: 2:215573795-2:217715661
1st Population
$-\log_{10}(p)$
3.5
3.0
2.5
2.0
1.5
1.0
0.5
PIP
[0.0, 0.2)
[0.2, 0.4)
[0.4, 0.6)
[0.6, 0.8)
[0.8, 1.0]
BARD1
LINC01963
SMARCAL1
IGFBP2
IGFBP5
−1.0
−0.5
0.0
0.5
1.0
